# Comparing the analgesic effects of ultrasound-guided caudal block and dorsal penile nerve block in pediatric concealed penis correction surgery: a randomized controlled trial

**DOI:** 10.3389/fped.2025.1607309

**Published:** 2025-07-24

**Authors:** Zhuopeng Lin, Yunhao Shao, Huidong Li, Zhifeng Chen, Yanfei Li, Shuhuan Wu, Nian Liu, Zhongqi Zhang

**Affiliations:** ^1^ Department of Anesthesiology, Shunde Heping Surgery Hospital, Foshan, China; ^2^ Department of Anesthesiology, The Affiliated Shunde Hospital of Jinan University, Foshan, China

**Keywords:** caudal block, dorsal penile nerve block, pediatric analgesia, regional anesthesia, concealed penis surgery

## Abstract

**Background:**

Effective perioperative analgesia is critical for pediatric patients undergoing concealed penis correction surgery. Despite the utility of regional techniques like caudal block (CB) and dorsal penile nerve block (DPNB), evidence comparing their efficacy in this population remains limited. This study aimed to compare ultrasound-guided CB and DPNB for perioperative analgesia in pediatric concealed penis surgery.

**Methods:**

In this prospective, double-blind, randomized controlled trial, 86 children (aged 5–12 years, ASA I–II) were allocated to CB (*n* = 44) or DPNB (*n* = 42) groups. All the children were induced with general anesthesia using propofol and sevoflurane, followed by laryngeal mask placement. Anesthesia was maintained with sevoflurane inhalation (spontaneous respiration preserved) under depth-of-anesthesia monitoring. The CB group received ultrasound-guided CB, while the DPNB group underwent bilateral dorsal penile nerve block, both using 0.2% ropivacaine. Primary outcomes included postoperative analgesic requirements within 24 h. Secondary outcomes encompassed pain scores, hemodynamic parameters, adverse events, and satisfaction.

**Results:**

The CB group demonstrated significantly lower analgesic requirements (38.6% vs. 71.4%, *p* = 0.005) within 24 h and reduced early postoperative pain scores (at 2,4,6 h, *p* < 0.05). Intraoperatively, CB required fewer anesthesia deepening (20.5% vs. 52.4%, *p* = 0.004) and shorter surgical duration (71.1 ± 13.7 vs. 79.7 ± 9.9 min, *p* = 0.001). Adverse events, including tachycardia (2.3% vs. 26.2%, *p* = 0.004) and body movements (6.8% vs. 42.9%, *p* < 0.001), were less frequent with CB. Parental and surgeon satisfaction were higher in the CB group (*p* = 0.049 and *p* < 0.001).

**Conclusions:**

Ultrasound-guided CB provides superior perioperative analgesia, fewer complications, and higher satisfaction compared to DPNB in pediatric concealed penis surgery, supporting its preference for this specific procedure in clinical practice.

**Clinical Trial Registration:**

https://www.chictr.org.cn/showproj.html?proj=178288, identifier ChiCTR2200065359.

## 1 Introduction

Effective perioperative pain management is crucial for pediatric patients undergoing surgery, as inadequate analgesia may prolong recovery, increase postoperative complications, parental anxiety and medical costs (1–3). The ideal anesthetic technique should provide optimal analgesia while minimizing risks. Although general endotracheal anesthesia effectively resolved intraoperative pain management, it inadequately meets postoperative analgesic demands. Thereby, many surgical procedures have gradually adopted regional anesthesia to improve postoperative pain management and patient recovery (4). Regional anesthesia techniques offer both intraoperative and postoperative analgesia, and their combination with general anesthesia intubation is particularly valuable (5). Despite significant advances in pediatric anesthesia, the selection of the most effective and safest regional anesthesia technique for these procedures remains controversial.

Caudal block (CB) and dorsal penile nerve block (DPNB) are two commonly used regional anesthetic techniques in pediatric urological surgery, each with distinct advantages and limitations (6, 7). CB provides extensive neural blockade covering the sacral dermatomes (S2–S4), which innervate the penis, perineum, and relevant visceral structures involved in penile surgery (8–10). This extensive coverage targets both somatic and visceral nociceptive pathways. Conversely, DPNB provides localized analgesia but often require bilateral blockade in pediatric patients with variable success rates (11, 12). The application of ultrasound-guided technology has improved the accuracy of these two anesthesia procedures, yet evidence comparing their intraoperative and postoperative analgesic efficacy in concealed penis correction surgery remains insufficient. Elucidating the analgesic effectiveness and safety of CB vs. DPNB could significantly improve pain management strategies for this vulnerable patient population.

This randomized controlled trial was designed to compare the perioperative analgesic efficacy between ultrasound-guided CB and DPNB in pediatric concealed penis surgery. This study evaluates perioperative safety, analgesic consumption, pain scores, and adverse event incidence to optimize pain management protocols for this common pediatric urological procedure, ultimately aiming to enhance clinical outcomes and patient satisfaction.

## 2 Methods

### 2.1 Study design

A prospective, randomized, double-blind study was conducted from January 2022 to September 2024. The study received approval from the Ethics Committee of the Shunde Heping Surgical Hospital (Approval No. HPYY-LL-2022001) and was registered with the Chinese Clinical Trial Registry (Registration No. ChiCTR2200065359). Informed consent was obtained from the family members of the children for this study.

### 2.2 The inclusion and exclusion criteria

Children scheduled to undergo pediatric concealed penis correction surgery.

The inclusion criteria: Age between 5 and 12 years old; confirmed diagnosis of concealed penis through clinical physical examination and imaging examinations such as ultrasound. American Society of Anesthesiologists (ASA) physical status classification of grade I–II.

Exclusion criteria: Those with severe cardiac, hepatic, or renal diseases; neurological disorders; a known allergy to anesthetic agents; mental health conditions or impaired cognitive function; a history of hemorrhagic disorders; or recent use of analgesics or sedatives within the past three months; fail to complete the required study observations, have incomplete records, or exhibit extremely poor compliance.

### 2.3 Grouping and blinding method

Children were randomized using a computer-generated random number table. Each patient was assigned a unique identification number and allocated to one of two groups based on the parity of the random number: patients with odd numbers were assigned to the CB group, while those with even numbers were assigned to the DPNB group. A double-blind design was used, with patients, parents, and the research team (surgeons and data collectors) unaware of group assignments. During the procedure, an independent anesthesia team performed the regional block, while the surgical team and the intraoperative/postoperative assessment team remained unaware of the group allocations. After the anesthesia procedure was completed, a separate intraoperative assessment team collected relevant data. Postoperative pain assessments were conducted by an independent nursing team, who were also blinded to the group assignments.

### 2.4 Anesthesia procedure

Upon entering the operating room, children underwent intravenous injection of propofol at a dose of 2 mg/kg and inhalational induction with sevoflurane, followed by laryngeal mask insertion. Anesthesia was maintained with sevoflurane inhalation while preserving spontaneous respiration. Standard monitoring was applied to all patients, including mean arterial pressure (MAP), heart rate (HR), oxygen saturation (SpO_2_), depth of anesthesia, body temperature, respiratory rate (RR), end-tidal carbon dioxide concentration (ETCO_2_), and end-tidal concentration of inhaled anesthetics.

For the CB group, patients were placed in the left lateral decubitus position with their legs flexed toward the abdomen to fully expose the sacral region. The skin around the sacral hiatus was routinely disinfected. Using an ultrasound machine equipped with a high-frequency linear array probe, the probe was placed along the midline of the dorsal sacrum to obtain a transverse view of the sacral hiatus. After identifying the sacral cornu and sacrococcygeal ligament, the probe was rotated 90° to obtain a longitudinal view. A 22G needle was inserted using an in-plane technique under direct ultrasound visualization. The needle tip position within the sacral canal was confirmed by both a distinct loss of resistance as it penetrated the sacrococcygeal ligament and real-time ultrasound imaging showing its path and final location. After negative aspiration for blood or cerebrospinal fluid, 0.2 ml/kg of 0.2% ropivacaine was injected. Ultrasound imaging confirmed cephalad spread of local anesthetic within the epidural space, targeting blockade of the S2–S4 nerve roots which innervate the penis and perineum. Patients were then placed supine.

For the DPNB group, patients were placed in the supine position with their legs abducted and slightly flexed to fully expose the penile region. The skin around the root of penis was routinely disinfected. Using an ultrasound machine equipped with a high-frequency linear array probe, the probe was placed transversely at the root of penis to obtain a clear transverse view of the urethral sponge, bulbospongiosus, dorsal artery and vein of penis, Buck's fascia, and tunica albuginea. The dorsal nerve of penis, located between Buck's fascia and the tunica albuginea and accompanying the dorsal artery of penis, appeared as a hypoechoic cord-like structure on the ultrasound image. A 22G block needle was inserted using an in-plane technique under ultrasound guidance, advancing from the lateral side of the root of penis toward the dorsal side. The needle was advanced through the hyperechoic superficial fascia, and a loss of resistance indicated that the needle tip had passed through the superficial fascia. The needle tip was then positioned between Buck's fascia and the tunica albuginea, adjacent to the dorsal artery of penis. After confirming the absence of blood upon aspiration, 0.2% ropivacaine was injected at a dose of 0.1 ml/kg. The same procedure was repeated on the contralateral side using an identical concentration and volume of local anesthetic.

Surgery began 20 min after block completion in both groups. Rescue anesthesia (propofol 1 mg/kg and fentanyl 1 μg/kg IV) was administered if body movement or a HR increase ≥20% from baseline occurred during skin incision, with repeat doses as needed.

### 2.5 Outcomes

1. The primary outcome was the proportion of patients requiring analgesics within 24 h postoperatively. Rescue analgesia (diclofenac sodium 25 or 50 mg rectally or intravenous ketorolac tromethamine 15 or 30 mg based on body weight) was administered if the resting pain score > 3 during this period.
2. Secondary outcome
  (1) Pain assessment: VAS scores (0 = no pain, 10 = severe pain) were recorded at 2, 4, 6, 12, 24, and 48 h postoperatively. For verbal children, self-reported pain levels were obtained. For non-verbal children, pain intensity was assessed using the FLACC (Face, Legs, Activity, Cry, Consolability) behavioral scale (13).
  (2) Surgical and anesthetic parameters: Surgery duration (duration from skin incision to wound closure), anesthesia time (duration from induction to the last administration of anesthetic agents), recovery time (duration from surgery completion to full awakening), anesthetic drug consumption (sevoflurane and propofol administered during the procedure). The number of cases in which the anesthesia needed to be deepened, as well as the MAP, HR, SpO_2_, RR were recorded before anesthesia (T1) and at the time of skin incision (T2).
  (3) Adverse events: Intraoperative and postoperative adverse events were documented, including tachycardia (HR increase >20% from baseline), intraoperative body movement (the child's body showed obvious involuntary movements, which affected the surgical operation), emergence agitation (restlessness, disorientation, or thrashing during recovery), postoperative nausea and vomiting (PONV), lower limb weakness, urinary retention, and wound complications (e.g., bleeding, edema, infection, and itching).
  (4) Satisfaction evaluation: Parents’ satisfaction was assessed 24 h postoperatively *via* a questionnaire administered by an independent anesthesia nurse. The questionnaire evaluated pain relief, recovery quality, and adverse events. Surgeons’ satisfaction was evaluated immediately post-surgery using a questionnaire focusing on patient cooperation and analgesic efficacy during the procedure. Both evaluations categorized satisfaction as “satisfied,” “partially satisfied,” or “unsatisfied” based on predefined criteria.

### 2.6 Statistical analysis

Sample size calculation: The primary observation indicator of this study was the proportion of children using analgesics within 24 h after surgery. The results of the preliminary trial showed that the proportion of children using analgesics within 24 h after surgery was 30% in the CB group and 60% in the DPNB group. After setting a significance level of 5% and a power of 80%, and fully taking into account a 5% dropout rate, the required sample size for each group was determined to be 44 cases.

The statistical analysis was performed using IBM SPSS Statistics (version 27.0). Continuous variables were expressed as mean ± standard deviation (SD) if normally distributed, or median (interquartile range [IQR]) if non-normally distributed. Normality of data distribution was assessed using the Shapiro–Wilk test. Categorical variables were described as frequency (percentage). Intergroup comparisons were conducted in the following ways: for continuous variables, independent *t*-tests were utilized for data with a normal distribution, while the Mann - Whitney U test was applied to non-parametric data. As for categorical variables, either the Chi-square test or Fisher's exact test was used, depending on the expected cell frequencies. *p* < 0.05 was considered statistically significant.

## 3 Results

### 3.1 Study flowchart and basic information of children

**Figure 1:**
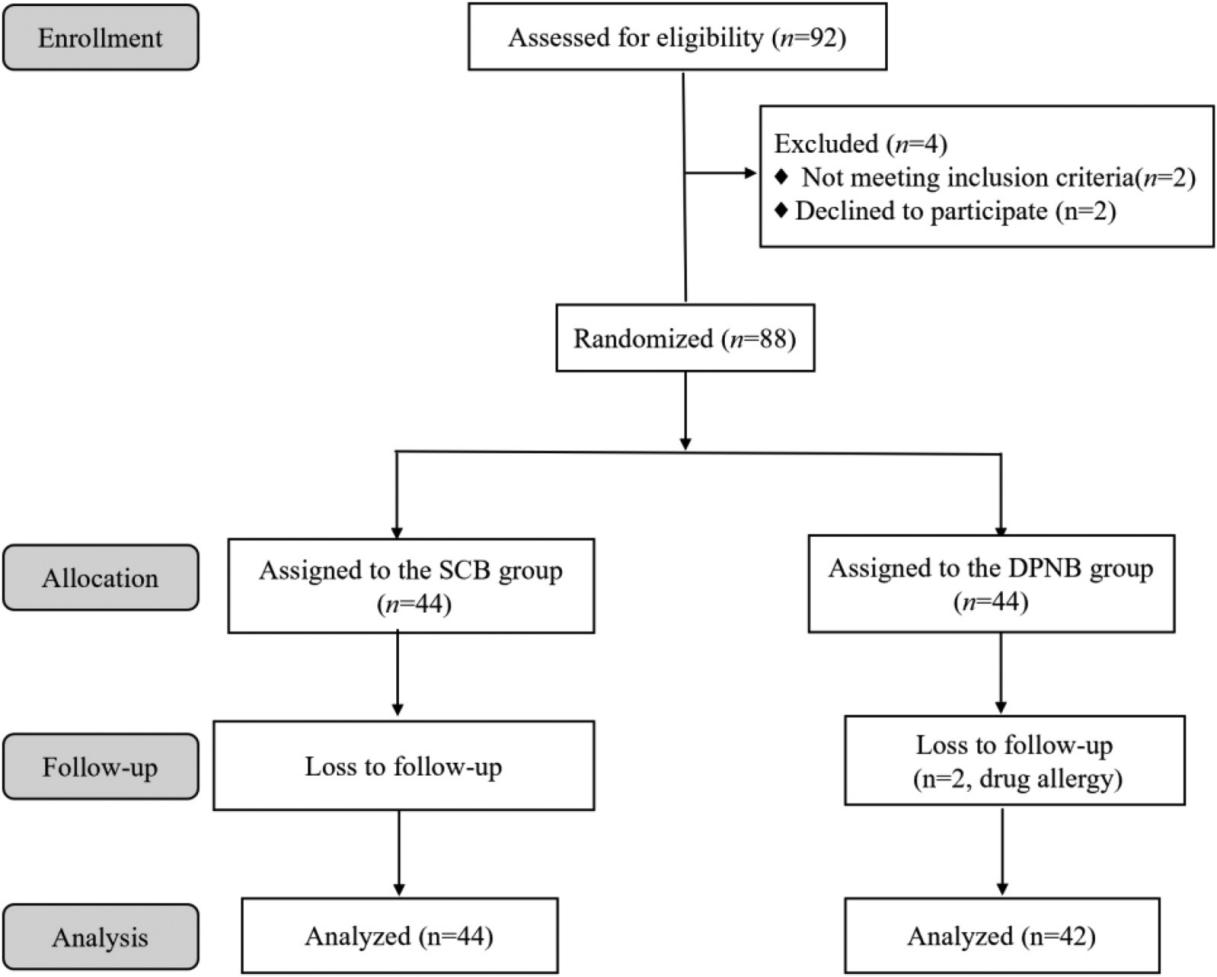
Study flowchart.

A total of 92 pediatric patients were initially assessed for eligibility, of whom 4 were excluded. Consequently, 88 patients were randomly assigned to either the CB or DPNB group, with 44 patients in each group. Two patients from the DPNB group were lost to follow-up due to drug allergies, resulting in a final sample size of 86 patients who completed the study. This included 44 patients in the CB group and 42 patients in the DPNB group, as shown in Figure 1. No significant differences in age, height, weight, or body mass index (BMI) between groups (all *p* > 0.05), as shown in Table 1.

**Table 1:** Patient characteristics.

Characteristics	CB group *n* = 44	DPNB group *n* = 42	*t*	*P*
Age (year)	8.6 ± 1.6	8.6 ± 1.6	0.064	0.963
Height (cm)	136.0 ± 11.6	137.8 ± 11.1	0.738	0.462
Weight (kg)	36.1 ± 9.0	37.2 ± 10.7	0.552	0.582
BMI	19.2 ± 3.0	19.3 ± 3.7	0.078	0.938

Data are expressed as mean ± SD.

BMI, body mass index.

### 3.2 Perioperative related indicators and satisfaction

CB group demonstrated a shorter surgery duration (*P* = 0.001), lower propofol consumption (*P* = 0.001), and fewer children of intraoperative anesthesia deepening (*P* = 0.004). Both parents and surgeons demonstrated significantly higher satisfaction with outcomes in CB group compared to DPNB group (*p* = 0.049 and *p* < 0.001, respectively). Other measures like anesthesia time, recovery time, and drug usage (sevoflurane) showed no significant differences, as shown in Table 2.

**Table 2:** Comparison of perioperative related indicators and satisfaction.

Item	CB group *n* = 44	DPNB group *n* = 42	*t*/*χ*^2^	*P*
Surgery duration (min)	71.1 ± 13.7	79.7 ± 9.9	3.340	0.001*
Anesthesia time (min)	101.5 ± 19.7	104.8 ± 15.4	0.865	0.390
Recovery time (min)	19.1 ± 8.7	22.1 ± 8.4	1.620	0.109
Sevoflurane (ml)	27.8 ± 8.6	30.9 ± 7.7	1.756	0.083
Propofol (mg)	222.9 ± 100.8	276.3 ± 99.6	3.153	0.001*
Anesthesia deepened intraoperatively [*n* (%)]	9 (20.5)	22 (52.4)	8.167	0.004*
Parents’ satisfaction (*n*)			–	0.049*
Unsatisfied	1	1		
Partially satisfied	5	13		
Satisfied	38	28		
Surgeons’ satisfaction (*n*)			–	<0.001*
Unsatisfied	1	6		
Partially satisfied	1	12		
Satisfied	42	24		

Data are expressed as mean ± SD or *n* (%).

*Indicates a statistical difference.

### 3.3 Postoperative pain scores and analgesic requirements

Significantly lower pain scores were observed in CB group compared to DPNB group at 2 h, 4 h, 6 h (*P* < 0.05). No significant differences were noted at 12, 24, or 48 h (*P* > 0.05). The mean VAS within 24 h was significantly lower in the CB group (*P* < 0.001). Additionally, the CB group demonstrated a reduced proportion of analgesic requirements within 24 h (38.6% vs. 71.4%, *p* = 0.005), as shown in Table 3.

**Table 3:** Comparison of postoperative pain scores and analgesic utilization between two groups.

Parameter	CB group *n* = 44	DPNB group *n* = 42	Z/*χ*^2^	*P*
At 2 h	0 (0,1.75 [0,4])	2 (0,3.25 [0,6])	2.822	0.005*
At 4 h	2 (0,2 [0,3])	2 (2,4 [0,6])	4.219	<0.001*
At 6 h	2 (2,3 [0,6])	4 (2,4 [0,6])	3.456	<0.001*
At 12 h	3 (2,3 [0,6])	2 (2,4 [0,6])	0.558	0.577
At 24 h	2 (0,2 [0,6])	2 (0,2 [0,5])	0.632	0.528
At 48 h	0 (0,2 [0,6])	0 (0,2 [0,2])	0.660	0.509
Mean VAS within 24 h	2.25 (1.5,3 [0,4])	3.5 (2.4,4 [0,5.5])	3.563	<0.001*
Analgesic requirements within 24 h	17 (38.6)	30 (71.4)	8.047	0.005*

Data are expressed as M (Q1, Q3 [Min–Max]) or *n* (%).

*Indicates a statistical difference.

### 3.4 The proportion of children with pain scores > 3 of postoperative

**Figure 2:**
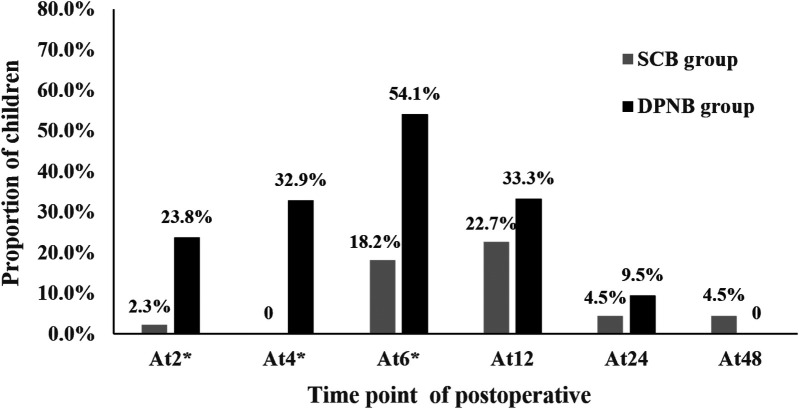
Proportion of children with pain scores > 3 at different time points of postoperative. *Indicates a statistical difference.

The proportion of children with clinically significant pain scores >3 demonstrated distinct patterns between the two groups across postoperative time points (Figure 2). Statistically significant intergroup differences were observed at 2 h (2.3% vs. 23.8%., *p* = 0.005), 4 h (0 vs. 32.9%, *p* < 0.001), and 6 h (18.2% vs. 54.1%, *p* = 0.001). The proportion of children with pain scores >3 reached a peak at 6 h. Subsequently, it gradually decreased over time. Moreover, at 48 h, the pain of the children in both groups was basically and completely relieved.

### 3.5 Hemodynamic changes during skin incision

The DPNB group exhibited significantly higher HR and RR during skin incision compared to CB group (*p* = 0.013 and *p* = 0.033, respectively). Intragroup analysis revealed a significant increase in HR and RR from T1 to T2 in the DPNB group (*p* < 0.05), while no significant change occurred in the CB group. MAP and SpO₂ levels showed no significant differences between groups, as shown in Table 4.

**Table 4:** Comparison of hemodynamic between the two groups at different time points.

Item	Time point	CB group *n* = 44	DPNB group *n* = 42	*t*	*P*
HR (beats/min)	T1	85.7 ± 10.3	87.4 ± 9.9	0.739	0.462
	T2	87.7 ± 11.3	95.3 ± 12.4^a^	2.546	0.013*
RR (breaths/min)	T1	21.6 ± 3.6	22.4 ± 3.5	1.040	0.302
	T2	21.7 ± 4.5	26.8 ± 4.8^a^	2.169	0.033*
MAP (mmHg)	T1	96.4 ± 9.8	95.8 ± 9.1	0.312	0.765
	T2	84.9 ± 10.6	88.5 ± 11.4	1.493	0.139
SpO_2_ (%)	T1	99.8 ± 0.3	99.7 ± 1.0	1.201	0.233
	T2	99.9 ± 0.3	99.8 ± 0.6	0.785	0.435

Data are expressed as mean ± SD.

HR, heart rate; RR, respiratory rate; MAP, mean arterial pressure; SpO₂, oxygen saturation; T1, before anesthesia; T2, at the time of skin incision.

^a^ Intragroup *P* < 0.05 vs. T1.

* Indicates a statistical difference.

### 3.6 Adverse events

More rate in the DPNB group (26.2%) than in the CB group (2.3%, *p* = 0.004) in tachycardia during skin incision. Higher in the DPNB group (42.9%) compared to the CB group (6.8%, *p* < 0.001) in intraoperative body movement. Other adverse reactions showed no significant differences between groups, as shown in Table 5.

**Table 5:** Comparison of adverse events between two groups.

Adverse events	CB group *n* = 44	DPNB group *n* = 42	*χ* ^2^	*P*
Tachycardia during skin incision	1 (2.3)	11 (26.2)	8.343	0.004*
Intraoperative body movement	3 (2.8)	18 (42.9)	13.232	<0.001*
Emergence agitation	2 (4.5)	1 (2.4)	–	>0.999
Excessive RR during surgery	9 (20.5)	11 (26.2)	0.140	0.708
Throat spasm	1 (2.3)	0	–	>0.999
PONV	1 (2.3)	0	–	>0.999
Fever	0	1 (2.4)	–	0.488
Lower limb weakness	0	0	–	>0.999
Urinary retention	0	0	–	>0.999
Wound complications: bleeding, edema, infection, and itching	13 (29.5)	11 (26.2)	0.011	0.915

Data are expressed as *n* (%).

* Indicates a statistical difference.

## 4 Discussion

This study demonstrates that ultrasound-guided CB provides superior perioperative analgesia compared to DPNB in pediatric concealed penis correction surgery. These conclusions are supported by the following findings: the CB group required fewer intraoperative anesthesia deepening interventions, exhibited significantly reduced postoperative analgesic requirements within 24 h, lower pain scores during early recovery, fewer complications, and higher satisfaction rates.

The superior efficacy of CB is anatomically grounded. It reliably blocks the sacral nerve roots (S2–S4), which carry both somatic sensory fibers (via the pudendal nerve) innervating the penile shaft and glans, and autonomic fibers (via the pelvic plexus) innervating deeper penile structures, the urethra, and bladder neck (8, 14–16). This dual blockade effectively addresses the somatic and visceral nociceptive input generated during concealed penis surgery (16, 17). In contrast, DPNB primarily targets the terminal somatic branches (dorsal nerves of the penis) and lacks significant visceral coverage (18–20). Our results confirm this broader blockade: CB significantly reduced intraoperative hemodynamic fluctuations (tachycardia, elevated HR/RR during incision) and body movements, indicating better suppression of the surgical stress response mediated via unblocked visceral pathways during DPNB. The lower proportion of patients requiring rescue analgesics within 24 h (38.6% vs. 71.4%) and significantly reduced early pain scores (2 h, 4 h, 6 h) in the CB group further reflect its more comprehensive and prolonged analgesic effect, likely encompassing both somatic incision pain and visceral discomfort from tissue manipulation and bladder/urethral traction.

Ultrasound guidance enhanced the precision and safety of both techniques in this study. For CB, real-time visualization allowed confirmation of needle placement within the sacral canal and monitoring of local anesthetic spread (21, 22), mitigating challenges posed by anatomical variations in the sacral hiatus (9, 15). Our technique utilized longitudinal and transverse views to confirm needle tip position post-loss-of-resistance, ensuring accurate drug delivery targeting the S2–S4 roots. The use of low-concentration ropivacaine (0.2%, 0.2 ml/kg) minimized motor block risks; no urinary retention or lower limb weakness occurred, consistent with sparing of higher sacral/lumbar roots responsible for leg movement and bladder voiding (23). Conversely, the higher incidence of tachycardia, movement, and subsequent propofol/fentanyl requirements in the DPNB group likely stem from incomplete blockade of deeper nociceptive pathways despite technically adequate bilateral somatic blocks under ultrasound (11, 24).

Postoperative wound complications (bleeding, edema, infection, itching) occurred in >26% of patients in both groups. It is essential to clarify that these complications are primarily determined by surgical technique and postoperative wound care, not by the choice of regional anesthetic technique (CB or DPNB). Other adverse events showed no significant differences. Higher parental satisfaction with CB (*p* = 0.049) reflects effective analgesia reducing caregiver anxiety (25, 26). Greater surgeon satisfaction (*p* < 0.001) is attributable to fewer intraoperative disruptions and smoother procedural flow with CB.

Limitations of this study warrant consideration. First, the single-center design and moderate sample size (*n* = 86) may limit generalizability. Second, while using validated FLACC and VAS scales, pain assessment remains inherently subjective, especially in preverbal children. Third, anatomical variations in the sacral canal (present in up to 20% of children) can challenge CB placement even with ultrasound, potentially impacting success rates (9, 15). Fourth, our findings are specific to concealed penis correction surgery; extrapolation to other pediatric urological procedures requires caution. Future research should include: (1) multicenter randomized trials with larger samples; (2) longer-term outcome assessment; (3) investigation of adjuvants (e.g., dexmedetomidine) to prolong CB duration; and (4) comparison with other advanced regional techniques like pudendal nerve block.

In conclusion, ultrasound-guided CB offers superior perioperative analgesia, fewer complications, and higher satisfaction compared to DPNB in pediatric concealed penis surgery. The extensive blockade of S2–S4 dermatomes effectively addresses both somatic and visceral pain components of this procedure. These findings support CB as the preferred regional anesthetic technique for this specific surgery, although broader recommendations await validation through multicenter studies.

## Data Availability

The original contributions presented in the study are included in the article/Supplementary Material, further inquiries can be directed to the corresponding author.
